# Patent Overview of Innovative Hyaluronic Acid-Based Hydrogel Biosensors

**DOI:** 10.3390/bios14120567

**Published:** 2024-11-24

**Authors:** Ahmed Fatimi, Fouad Damiri, Mohammed Berrada, Adina Magdalena Musuc

**Affiliations:** 1 Chemical Science and Engineering Research Team (ERSIC), Department of Chemistry, FPBM, Sultan Moulay Slimane University (USMS), Mghila Campus, Beni Mellal 23000, Morocco; 2 Laboratory of Biomolecules and Organic Synthesis (BIOSYNTHO), Department of Chemistry, Faculty of Sciences Ben M’Sick, University Hassan II of Casablanca, Casablanca 20000, Morocco; fouad.damiri@ulb.be (F.D.); mohammed.berrada@univh2c.ma (M.B.); 3 Institute of Physical Chemistry—Ilie Murgulescu, Romanian Academy, 202 Spl. Independentei, 060021 Bucharest, Romania; amusuc@icf.ro

**Keywords:** hyaluronic acid, hydrogel, biosensor, innovation, patent analysis

## Abstract

Hyaluronic acid-based hydrogels are emerging as highly versatile materials for cost-effective biosensors, capable of sensitive chemical and biological detection. These hydrogels, functionalized with specific groups, exhibit sensitivity modulated by factors such as temperature, pH, and analyte concentration, allowing for a broad spectrum of applications. This study presents a patent-centered overview of recent advancements in hyaluronic acid hydrogel biosensors from 2003 to 2023. A total of 50 patent documents—including 41 patent applications and 9 granted patents—reveal a growing interest, primarily driven by United States-based institutions, which account for approximately 54% of all filings. This trend reflects the strong collaboration between universities, industry, and foundations in pushing this technology forward. Most patented technologies focus on biosensors for in vivo blood analysis, measuring critical parameters such as gas concentration and pH, with particular emphasis on glucose monitoring via tissue impedance using enzyme-immobilized oxidase electrodes. Additionally, the 9 granted patents collectively showcase key innovations, highlighting applications from continuous glucose monitors to implantable vascular devices and sweat analyte detection systems. These patents underscore the adaptability and biocompatibility of hyaluronic acid hydrogels, reinforcing their role in enhancing biosensor performance for real-time health monitoring. In summary, this overview highlights the importance of patent analysis in tracking and directing research and development, helping to clarify the field’s evolution and identify innovation gaps for hyaluronic acid-based hydrogel biosensors.

## 1. Introduction

**Figure 1:**
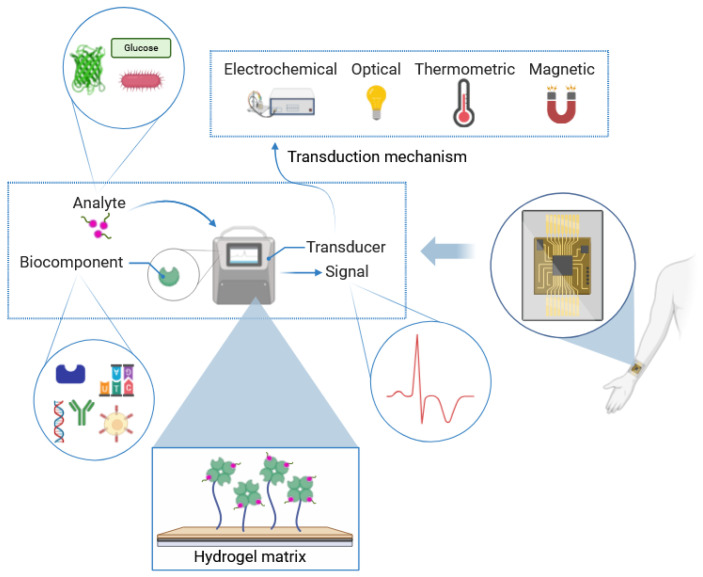
Schematic representation of hydrogel-based biosensors: composition, principle, and transduction mechanism. (Created with Biorender.com).

Hydrogel-based biosensors show promising potential for various applications, including biomedical applications, disease diagnosis, and detecting and quantifying pharmaceuticals [1,2]. These biosensors employ different detection principles (Figure 1). More specifically, the hydrogel matrix consists of a network of interconnected polymer chains that maintains the hydrated nature of the gel. Within or on the surface of this hydrogel network, specific biomolecules known as bioreceptor components are present (e.g., enzymes, antibodies, DNA, etc.). The target analyte, which is the molecule or pathogen to be detected (e.g., glucose, proteins, bacteria, etc.), interacts with these bioreceptors. The target analyte approaches and interacts with the bioreceptor component within or on the surface of the hydrogel network. This interaction forms the basis of the detection process. Depending on the type of sensor—whether optical, electrochemical, thermometric, magnetic, etc.—the biological recognition event is converted into a measurable signal [2,3,4]. Hyaluronic acid (HA)-based hydrogels with specific functional groups have been used as cost-effective, miniature biosensors for chemical and biological detection, with sensitivity dependent on factors such as temperature, pH, and concentration of analytes [2,3]. Thanks to the chemical structure of HA (detailed below), the target analyte approaches and interacts with the bioreceptor component within or on the surface of the HA hydrogel network [5]. Further, in HA-based hydrogel biosensors, different detection principles could be employed in which the biological recognition event is converted into a measurable signal, as shown in Figure 1.

Interest in HA, also known as hyaluronan, as a biomaterial has increased significantly since the early 1980s, with major clinical applications in ophthalmology (e.g., eye surgery), treatment of degenerative joint disease (e.g., viscosupplementation for arthritis), and prevention of adhesions (e.g., anti-adhesive component in plastic surgery), associated with large-scale production of the polymer [6]. HA offers several unique advantages as a starting material for obtaining hydrogels for regenerative medicine [7]. Firstly, it has a ubiquitous distribution in nature, found in virtually all animal species, in the capsule of some microorganisms, and in all human body tissues. Additionally, the repeating disaccharide unit of HA is identical across all species and tissues, making it immunologically non-foreign to its respective host [8]. HA is a naturally occurring biopolymer present in the extracellular matrix (ECM), primarily in cartilage, umbilical cord, synovial fluid, and skin. It is a linear, unbranched polysaccharide considered the main constituent of glycosaminoglycans (GAGs) and cartilage [9].

**Figure 2:**
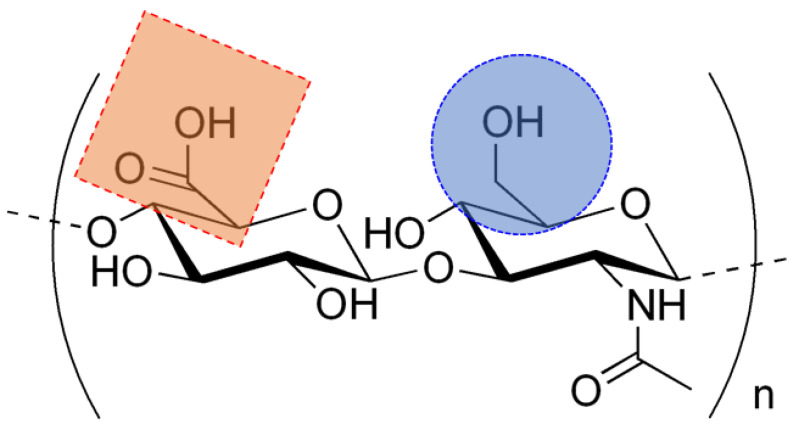
The chemical structure of HA (native) in its repeating disaccharide unit highlights the chemical groups targeted for chemical derivation: carboxylic groups (red dotted square) and secondary hydroxyl groups (blue dotted circle). (Created with ACD/ChemSketch (Freeware) 2023.1.2).

Naturally, HA helps protect joints by increasing synovial fluid viscosity and making cartilage more elastic [10]. HA is an anionic copolymer with different molecular weights (103 kDa < Mw < 104 kDa) and a simple chemical structure consisting of β-D-glucuronic acid and N-acetyl-β-D-glucosamine, linked by alternating glycosidic bonds (1 → 4) and (1 → 3) [9,11]. Its chains can reach lengths of 2000 to 5000 dimers [12]. Figure 2 presents the chemical structure of native HA in its repeating disaccharide unit and highlights the chemical groups targeted for chemical derivation. This structure allows for various modifications and applications in biological fields [13].

In recent years, the utilization of HA in biosensor technology has evolved significantly. Researchers have developed innovative biosensors utilizing HA-based materials for various applications. Studies have demonstrated the creation of HA-based electroconductive hydrogels for non-enzymatic glucose detection, showcasing high sensitivity, selectivity, and stability [14]. Additionally, HA has been employed in the design of immune devices for the rapid and sensitive recognition of HA in human plasma samples, offering a novel technique for HA analyses with a linear response and low detection limits [15]. Furthermore, the development of label-free electrochemical immunosensors based on modified ITO-PET (indium tin oxide-polyethylene terephthalate) electrodes has shown excellent reproducibility, selectivity, and stability for HA detection in real samples, indicating its potential as a diagnostic tool in clinical bioassays [16]. Moreover, a novel enzyme-responded controlled-release electrochemical biosensor has been constructed for hyaluronidase detection, providing a method for detecting hyaluronidase activity in urine samples with high sensitivity and a wide detection range [17]. Lastly, a thin film composed of HA hydrogel has been utilized in a filtration system for hyaluronidase detection, offering a simple yet sensitive method for detecting hyaluronidase in vitreous samples [18]. These advancements highlight the versatility and importance of HA hydrogels in the development of biosensors for various biomedical applications.

In this study, the advances in the development of HA-based hydrogel biosensors are addressed for the period 2003–2023. The publication years of patent documents in this area are presented, as well as patent jurisdictions and patent classification. Finally, a section dedicated to review of granted patents on innovative HA-based hydrogel biosensors is proposed.

## 2. Resources and Methods

The Lens Patent Data Set [19] and Google Patents Research Data [20] have been used according to different keywords related to the topic “hyaluronic acid-based hydrogel biosensors”. Patent documents were filtered to include only patent applications and granted patents published during the period 2003–2023. Analysis was then performed according to publication year, jurisdiction, and classification. For the review part, nine relevant granted patents have been selected from the found patent documents and reviewed based on innovative HA-based hydrogel biosensors.

## 3. Patent Analysis of Innovative HA-Based Hydrogel Biosensors

**Figure 3:**
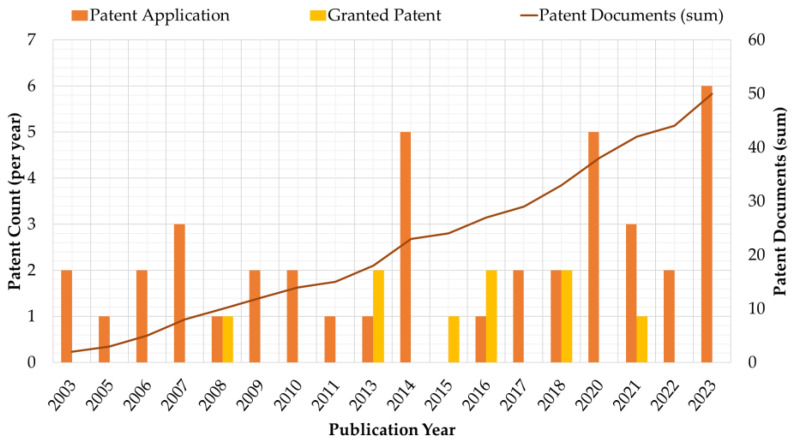
Publication year of patent documents (i.e., patent applications and granted patents) related to HA-based hydrogel biosensors from 2016 to 2023.

From 2003 to 2023, 50 patent documents have been published, with 50% of patent documents having been published from 2016 to 2023 (Figure 3). Further, the number of patents filed globally has increased five-fold from 2008 to 2023, owing to rapid developments and the pressing need for innovations in HA-based hydrogel biosensors. However, of the 50 patent filings, only nine patents have been granted.

**Figure 4:**
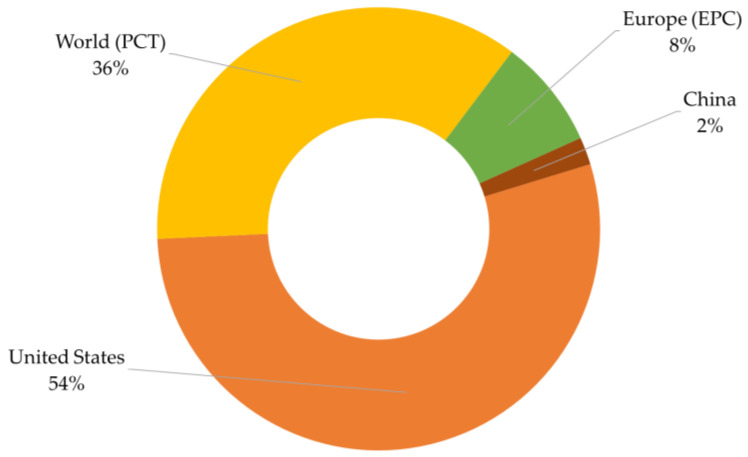
Jurisdictions of patent filing in relation to HA-based hydrogel biosensors from 2003 to 2023. The EPC is an international treaty managed by the EPO, establishing a single patent system that covers multiple European countries. The PCT is an international treaty administered by the WIPO, providing a unified procedure for filing international patent applications in multiple countries.

The jurisdictions for patent filings for HA-based hydrogel biosensors are shown in Figure 4. A patent jurisdiction defines the particular legal or geographical realm where a patent application is submitted or approved.

Jurisdiction in the context of patents can be understood at three levels: national, regional, and international. National jurisdiction refers to the legal authority of a single country to grant patents within its borders and enforce patent rights within that country [21]. Regional jurisdiction refers to the authority of a regional organization to grant patents that are valid across multiple countries within a defined geographic region. For example, the European Patent Office (EPO) grants European patents that are valid in its member states under the European Patent Convention (EPC) [22]. While international jurisdiction refers to mechanisms that facilitate the filing and coordination of patent applications across multiple countries, often through international treaties and agreements [23]. The Patent Cooperation Treaty (PCT) allows applicants to file a single international patent application that can be recognized in many countries worldwide, delaying the decision on where to seek national patent protection. The PCT is administered by the World Intellectual Property Organization (WIPO) [24].

Between 2003 and 2023, the United States recorded 27 patent documents, which account for over 54% of the patents discussed. Contributions from the PCT, the EPC, and China amount to 18, 4, and 1 patent filings, with 36, 8, and 1%, respectively. This reflects the United States’ leading role in developing technologies for HA-based hydrogel biosensors and their applications in the biomedical field.

Once a patent document (i.e., patent application or granted patent) is published, it is assigned a classification code based on the patent classification [25]. Among them, the Cooperative Patent Classification (CPC) is a globally used patent classification system that categorizes patents according to their technical features. The CPC system was jointly developed by the European Patent Office (EPO) and the United States Patent and Trademark Office (USPTO) [26]. It is hierarchically structured into main groups, subgroups, and sections. Each CPC code consists of a combination of numbers and letters that denote specific technologies covered by the patent. CPC codes are utilized by patent offices worldwide to classify and search for patents in specific technical fields. They facilitate efficient retrieval of patent documents and aid in assessing the patentability of inventions by identifying relevant prior art [27].

In relation to the subject of this paper, Table 1 provides the top 10 CPC codes for HA-based hydrogel biosensors. These classifications provide a structured way to categorize inventions based on their technical features and applications within various domains, such as medicine and chemistry.

Some commonly occurring CPC codes address the use of HA hydrogel improvement in common measuring for diagnostic purposes (e.g., A61B5/1486, A61B5/14532, A61B5/14865, etc.), devices or systems enabling patient monitoring using sensors (e.g., A61B2562/125), while others are aimed at medicinal preparations characterized by special physical form (e.g., A61B5/1473) and materials for prostheses or for coating prostheses (e.g., A61K47/61) [28,29,30,31,32].

Of the 50 entries, eight were classified into the use of HA hydrogels in devices or methods for measuring characteristics of blood in vivo (e.g., gas concentration, pH value, etc.) using enzyme electrodes (i.e., A61B5/1486). These eight patent documents involve advanced methods and devices for measuring various characteristics of blood directly within the body using enzyme electrodes. These electrodes often feature immobilized enzymes such as oxidase, which can interact with specific blood components (e.g., glucose, lactate, etc.) to generate a measurable signal. This technology is used to determine critical blood parameters like gas concentrations (e.g., oxygen, carbon dioxide, etc.) and pH values [33]. The high score indicates that this method is particularly effective and valuable, likely offering high accuracy and real-time monitoring capabilities, which are crucial for managing medical conditions and ensuring patient safety.

On the other hand, seven patent documents concern the use of HA hydrogels in instruments for measuring the characteristics of body fluids or tissues for measuring glucose by tissue impedance measurement (i.e., A61B5/14532). The found patent document focuses on devices and methods for measuring glucose levels in body fluids or tissues. One technique involves tissue impedance measurement, which evaluates the resistance of tissues to an electrical current, providing information on glucose concentration [34]. This non-invasive or minimally invasive approach is significant for continuous glucose monitoring, especially for diabetic patients [35]. The score reflects its substantial relevance and utility in providing real-time glucose monitoring, thereby aiding in effective diabetes management.

Similarly, seven patent documents concerned measuring characteristics of blood in vivo (e.g., gas concentration, pH value, etc.) using invasive enzyme electrodes (e.g., with immobilized oxidase, etc.) introduced into the body by a catheter or needle or using implanted sensors (i.e., A61B5/14865). This involves the use of invasive enzyme electrodes for measuring blood characteristics directly within the body. These electrodes, featuring immobilized enzymes such as oxidase, are introduced via a catheter, needle, or implanted into the body to continuously monitor parameters like gas concentrations and pH values [36,37]. Despite being invasive, this method provides highly accurate and continuous data that is critical for patients requiring intensive monitoring, such as those in critical care or with chronic conditions [38]. The score indicates that while it is highly effective and valuable, its invasiveness may limit its widespread use compared to non-invasive methods.

Other CPC codes in the top 10 include also 3 other domains of HA-based hydrogel biosensor formulations, such as devices or systems enabling patient monitoring using sensors and medicinal preparations characterized by special physical forms and materials for prostheses or coating prostheses, highlighted with A61B2562/125, A61B5/1473, and A61K47/61, respectively.

**Table 1:** Top 10 CPC codes as a function of patent count and HA-based hydrogel applications/formulations in the biosensor area.

CPC Code	Domaine	HA-Based Hydrogel Applications/Formulations *	Patents (Count)
A61B5/1486	Measuring for diagnostic purposes	Measuring characteristics of blood in vivo (e.g., gas concentration, pH value) using enzyme electrodes (e.g., with immobilized oxidase)	8
A61B5/14532	Measuring for diagnostic purposes	Measuring the characteristics of body fluids or tissues for measuring glucose (e.g., by tissue impedance measurement)	7
A61B5/14865	Measuring for diagnostic purposes	Measuring characteristics of blood in vivo (e.g., gas concentration, pH value) using invasive enzyme electrodes (e.g., immobilized oxidase) introduced into the body by a catheter or needle or using implanted sensors	7
A61B2562/125	Devices or systems enabling patient monitoring using sensors	Manufacturing methods specially adapted for producing sensors for in vivo measurements characterized by the manufacture of electrodes	6
A61B5/686	Measuring for diagnostic purposes	Permanently implanted devices (e.g., invasive sensors, pacemakers, other stimulators, biochips, etc.)	6
A61B5/14546	Measuring for diagnostic purposes	Measuring characteristics of blood in vivo (e.g., gas concentration, pH value) for measuring analytes (e.g., ions, cytochromes, etc.)	5
A61B5/1473	Medicinal preparations characterized by special physical form	Measuring characteristics of blood in vivo (e.g., gas concentration, pH value, etc.) using invasive chemical or electrochemical methods (e.g., by polarography) introduced into the body by a catheter	5
A61K47/61	Materials for prostheses or for coating prostheses	Medicinal preparations characterized by the non-active ingredients used, such as the organic macromolecular compound being a polysaccharide or a derivative thereof	5
A61K47/6903	Measuring for diagnostic purposes	Medicinal preparations characterized by the non-active ingredients used (e.g., a polysaccharide or a derivative thereof) bound to the active ingredient in the form of a semi-solid, such as a gel, a hydrogel, or a solidifying gel	5
A61L27/54	Measuring for diagnostic purposes	Materials for grafts or prostheses or for coating grafts or dental prostheses characterized by their function or physical properties, such as biologically active materials (e.g., therapeutic substances)	5

* These descriptions are adapted from the Espacenet Patent Search [39].

## 4. Review of Granted Patents on Innovative HA-Based Hydrogel Biosensors

Recent advancements in the field of biosensors have highlighted the significant potential of HA-based hydrogels. These biocompatible materials provide an ideal matrix for the development of sensitive and reliable biosensors capable of monitoring various analytes in biological fluids. This review covers nine granted patents, each presenting unique innovations in the application of HA hydrogels for biosensors. These patents collectively underscore the versatility and effectiveness of HA hydrogels in creating next-generation biosensors for continuous health monitoring, non-invasive diagnostics, and early disease detection (Table 2).

### 4.1. HA-Based Chemically Crosslinked Hydrogels for Biocompatible and Long-Term Glucose Sensing

**Figure 5:**
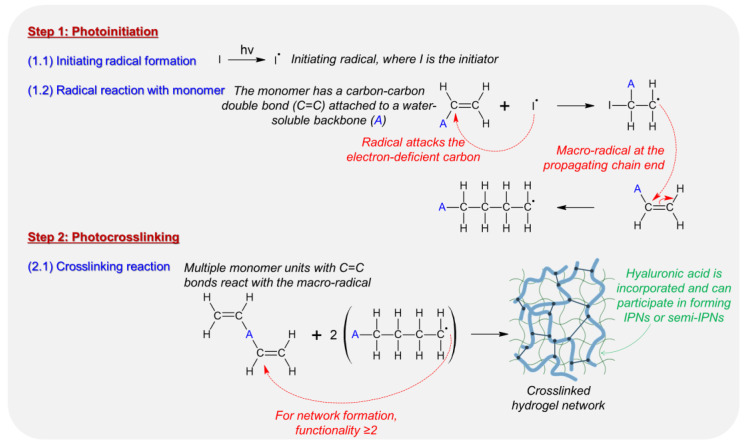
Photocrosslinking scheme summarizing the fundamental chemical reactions involved in the formation of a hydrogel network through light-induced polymerization and crosslinking. The crosslinked network is essential for the structural integrity and functionality of the hydrogel in applications such as the biosensor described in the patent. The process begins with photoinitiation, where the initiator (I) absorbs light energy (hν) and undergoes a splitting reaction to form a reactive radical (I^•^). This radical formation is crucial as it initiates the subsequent reactions leading to polymerization. The radical then reacts with the monomer, which features a carbon-carbon double bond (C=C) attached to a water-soluble backbone (A). The scheme was adapted and designed from raw data presented in patent US8367109B2 [40]. (Created with ACD/ChemSketch Freeware).

In 2008, inventors Barman et al. claimed through their invention the innovative use of HA in hydrogel formulations for biosensors [40]. The described transdermal biosensor features a hydrogel that integrates an electrode and a biologically active enzyme to enable glucose detection. The hydrogel is created from a macromer solution that includes polyethylene glycol diacrylate (PEGDA), a hydrophilic polymer with cross-linkable end groups, a photoinitiator (such as Irgacure 2959 that enables free-radical polymerization when exposed to ultraviolet (UV) light), and glucose oxidase, which is essential for converting glucose into a detectable signal. Figure 5 shows the photoinitiation and photocrosslinking steps involved in constructing the crosslinked polymer network. With a macromer concentration exceeding 10% (*w*/*v*) and an optimized molecular weight, the solution forms a mesh-like network that effectively entraps the enzyme, ensuring stability and functionality within the biosensor. According to patent US7432069B2, the crosslinking process is initiated by UV exposure at a wavelength of approximately 365 nm. The UV light activates the photoinitiator, producing highly reactive free radicals that attack the electron-dense diacrylate groups within the PEGDA micelle cores. This initiates a crosslinking reaction that propagates outward through chain propagation, eventually stabilizing the structure into a hydrogel matrix. In other words, during UV exposure, PEGDA undergoes free-radical water-dispersed polymerization. Initially, the free radicals interact with the hydrophobic diacrylate groups within the core of the PEGDA micelles, facilitating a localized and controlled polymerization process. This ensures the bioactive enzyme glucose oxidase remains intact and functional within the hydrogel matrix, as the reaction’s exothermic properties are minimized by the aqueous environment that disperses any heat produced. The network density of the hydrogel, and thus the rate of enzyme release, can be adjusted by modifying the PEGDA concentration, molecular weight, or initiator levels. Furthermore, HA is incorporated into the hydrogel composition as a polymeric excipient, aiming to enhance properties such as water-absorbing and water-retaining capabilities, gel strength, and the modulable release characteristics of the incorporated protein. This integration of HA into the hydrogel matrix significantly contributes to the overall effectiveness and functionality of the biosensors. Additionally, HA can participate in forming interpenetrating networks (IPNs) or semi-interpenetrating networks (semi-IPNs) with the PEGDA macromer, significantly contributing to the structural integrity and performance of the biosensors. HA is considered one of the key polymers that can participate in these network formations. This novel use of HA hydrogels offers improved structural and release properties, making them highly suitable for advanced biosensing applications [40].

### 4.2. HA Hydrogel-Based Encapsulation of Microbes for Advanced Biosensing Applications

**Figure 6:**
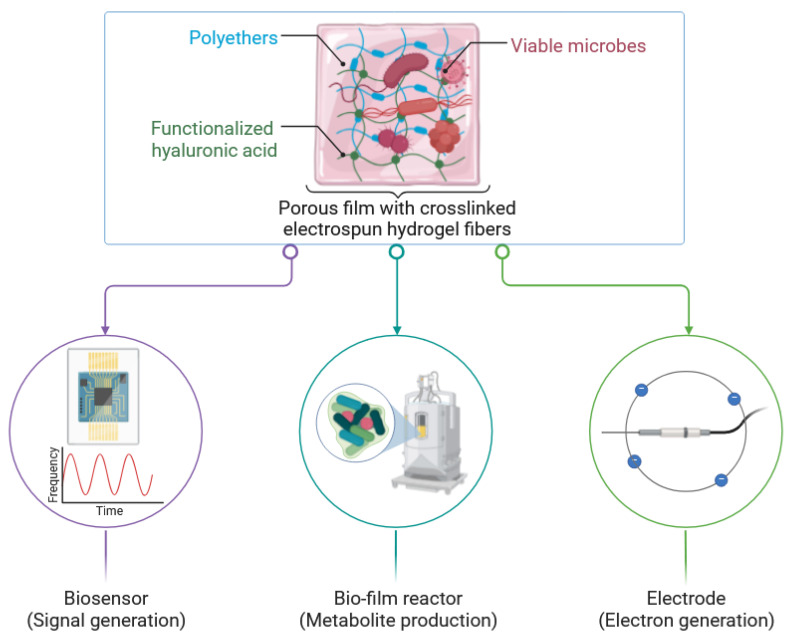
Schematic illustration of the invented porous film structure integrated with crosslinked hydrogel fibers containing encapsulated microbes. The hydrogel fibers are made from a combination of polyethers and functionalized HA. The invention is intended for functional applications such as biosensors, biofilm reactors, or electrodes. Scheme was created and designed from raw data presented in patent US8367109B2 [41]. (Created with Biorender.com).

In 2013, inventors Chidambaram et al. claimed through the invention described in patent US8367109B2 the novel use of HA hydrogel for biosensors [41]. This invention centers around the encapsulation of viable microbes within crosslinked electrospun hydrogel fibers, made from a combination of polyethers (e.g., polyethylene oxide, polypropylene oxide, etc.) and functionalized HA, where HA serves as a significant component due to its biocompatibility and ability to be modified for crosslinking. The patent details that the HA can be functionalized with crosslinkable groups through a crosslinking process likely involving agents such as ammonium persulfate to form a stable hydrogel network capable of encapsulating microorganisms. This hydrogel network maintains the viability of the encapsulated microbes while rendering the fibers insoluble and permeable. The crosslinked electrospun hydrogel fibers created in this manner can be used in biosensors that detect specific chemical compounds by generating a detectable signal, often an electrical one, in response to the presence of those compounds (Figure 6). Moreover, the invention emphasizes the utility of these biosensors in various applications, including environmental monitoring and medical diagnostics. By incorporating HA into the hydrogel matrix, the biosensors benefit from its natural abundance, low immunogenicity, and excellent water retention properties, making them highly effective for maintaining microbial viability and functionality within the sensor framework [41].

### 4.3. HA-Based Hydrogel Biosensor for Continuous Metabolite and Protein Monitoring: Design and Manufacturing Methods

**Figure 7:**
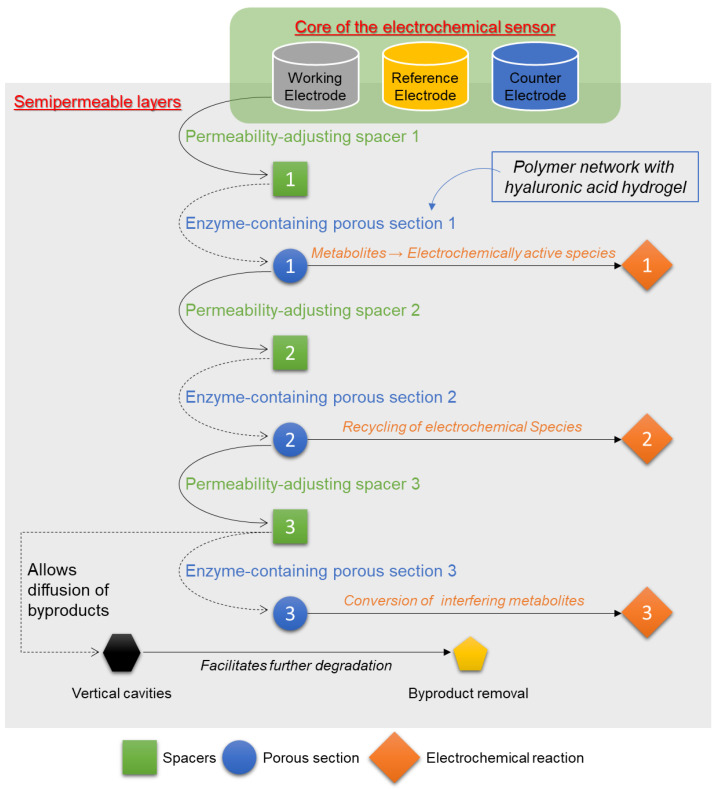
Schematic diagram representing the layers and components of the biosensor as described in the patent. This includes the electrodes, enzyme-containing porous sections, permeability-adjusting spacers, and the layers where the HA hydrogel plays a role thanks to its permeability properties and its contribution to controlling diffusion and facilitating reactions. The scheme was created and designed from raw data presented in patent US8608922B2 [42].

In 2013, inventors Papadimitrakopoulos et al. claimed through the invention detailed in patent US8608922B2 a biosensor for continuous monitoring of metabolites and proteins the use of various materials, including HA hydrogel, to improve the functionality and longevity of the biosensor [42]. The biosensor comprises a substrate, a reference electrode, a working electrode, a counter electrode, and a plurality of permeability-adjusting spacers. These components are arranged to create enzyme-containing porous sections. HA hydrogel, known for its biocompatibility and capacity to form hydrogels, is utilized within these enzyme-containing sections to ensure the stable and effective immobilization of enzymes, thereby enhancing the biosensor’s performance in detecting target metabolites (Figure 7). The use of HA hydrogel in biosensors addresses several critical issues related to sensor stability and longevity. Traditional biosensors often face challenges such as enzyme leaching and loss of activity over time. By incorporating HA hydrogel, which forms a stable, hydrated network, the enzymes remain more effectively immobilized, maintaining their activity and thus improving the accuracy and reliability of the sensor. This hydrogel also helps in managing the diffusion of analytes and byproducts within the sensing environment, ensuring that the enzymatic reactions occur efficiently and that the sensor readings remain precise over extended periods. Additionally, HA hydrogel provides a biocompatible interface between the biosensor and the biological environment in which it is implanted. This compatibility minimizes adverse immune responses and inflammation, which can otherwise degrade the sensor’s performance. The hydrogel’s structural properties allow it to conform to the tissue, reducing mechanical mismatch and enhancing the sensor’s integration with the host tissue. This integration is crucial for continuous monitoring applications, where long-term sensor functionality and stability are paramount [42].

### 4.4. HA-Based Implantable Vascular Biosensor with Engineered Capillary Beds for Real-Time Blood Analyte Monitoring

**Figure 8:**
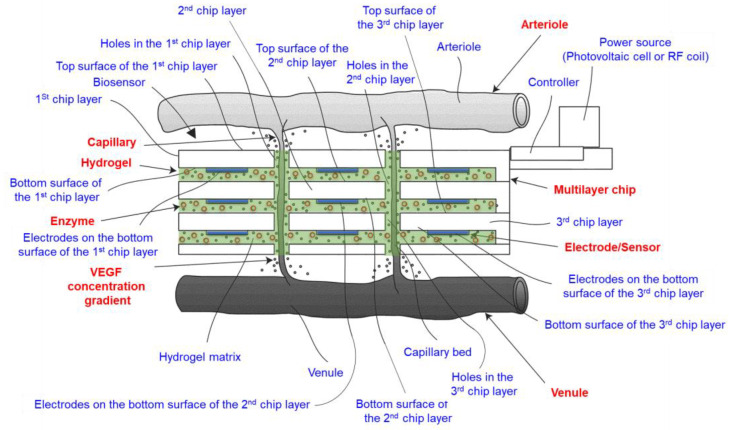
Schematic perspective view of the invented implantable biocompatible biosensor that incorporates several novel elements according to the claimed invention through the presented patent. The invention is particularly novel in how it leverages the body’s natural processes, such as angiogenesis, to enhance the functionality of a biosensor, making it potentially more accurate and integrated than traditional implantable sensors. The hydrogel matrix can be based on various materials, including, among others, HA. The scheme was adapted and designed from raw data presented in patent US9011330B [43].

In 2015, inventors Sadek et al. claimed through their invention a novel use of HA hydrogel for biosensors [43]. The patent US9011330B2 describes an implantable biocompatible biosensor designed to interface with the vascular system by stimulating the growth of organic material through a hydrogel matrix. This matrix fills micromachined holes in chip layers, promoting the formation of a capillary bed around the biosensor, which enables real-time monitoring of various analytes in the blood. The biosensor’s hydrogel matrix, which can be HA-based, incorporates angiogenesis-stimulating factors such as vascular endothelial growth factor (VEGF). When implanted, these factors diffuse from the hydrogel, inducing the formation of new blood vessels and capillaries. This growth allows blood to flow through the hydrogel, enabling analytes to diffuse into the matrix and be detected by on-chip sensors (Figure 8). The hydrogel matrix not only acts as a scaffold for new capillary growth but also serves as a medium for detecting biochemical reactions. It can incorporate biosensing molecules such as enzymes, antibodies, antigens, and catalysts. Upon binding primary analytes, these biosensing molecules produce secondary species detectable by the sensor array, thus providing accurate and continuous biochemical monitoring. This invention highlights the versatility and effectiveness of HA-based hydrogels in creating a biocompatible environment for implantable biosensors. The hydrogel’s ability to stimulate tissue growth and facilitate real-time analyte detection makes it a significant advancement in the field of biosensing technologies [43].

### 4.5. HA-Based Hydrogels for Enhanced Sensitivity in Biosensor Technologies

**Figure 9:**
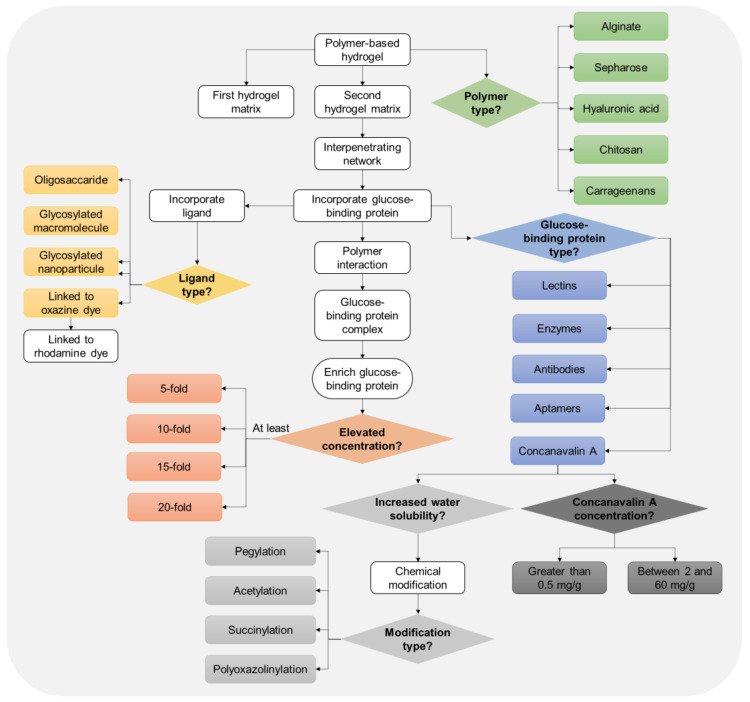
Flowchart representation of the invented method for enriching a dissolved glucose-binding protein in a hydrogel. This schematic visually represents the key components and interactions described in the invention, with a focus on the role of the HA hydrogel (among others), as the primary matrix, in enriching the glucose-binding protein. Flowchart was created and designed from raw data presented in patent US9244064B2 [44].

In 2016, inventors Miller et al. claimed through the invention of a biosensor that HA hydrogel plays a crucial role in the development of biocompatible and effective biosensors [44]. The patent US9244064B2 describes the use of HA-based hydrogel, among other materials, as a matrix that fills the plurality of holes in the sensor chip layers. This hydrogel matrix is critical for ensuring biocompatibility and stability of the implanted biosensor. The hydrogel matrix incorporating HA can also include angiogenesis-stimulating factors that promote the growth of capillaries into the sensor chip. This facilitates efficient diffusion and exchange of analytes between the capillaries and the sensor array within the chip. By acting as a buffer, the HA hydrogel matrix helps to minimize transient noise in the sensor signals, thereby enhancing the accuracy and reliability of the biosensor measurements. Furthermore, the HA hydrogel matrix can be combined with various biosensing molecules, such as enzymes, antibodies, antigens, and catalysts. These biosensing molecules can bind to primary analytes of interest, producing secondary species that are detectable by the on-chip electronic sensors (Figure 9). The inclusion of HA in the hydrogel matrix is particularly advantageous due to its inherent biocompatibility and capacity to support tissue integration and sensor functionality [44].

### 4.6. 3D HA Gydrogel Scaffolds with Covalently Immobilized Protein Gradients for Biosensing

**Figure 10:**
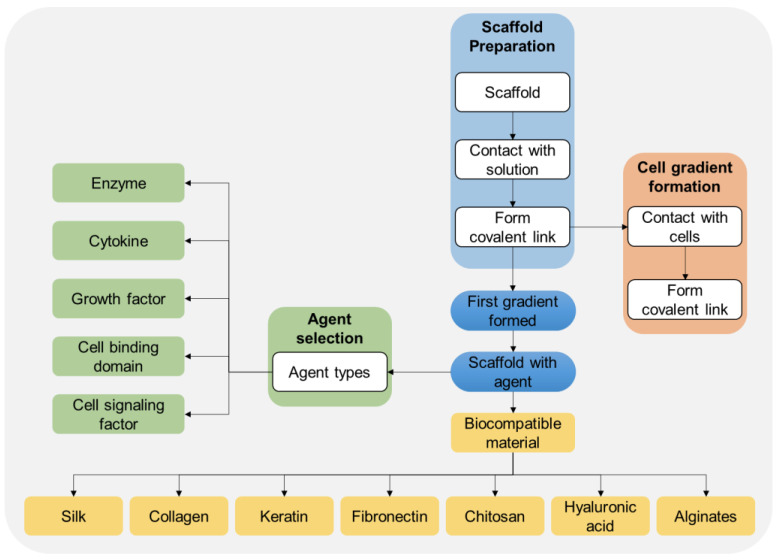
Flowchart representation of the methods described in the invention for forming immobilized agents and cell gradients within a 3D porous scaffold, particularly focusing on the use of HA (among others) as a biocompatible material. The flowchart was created and designed from raw data presented in patent US9290579B2 [45].

In 2016, inventors Vepari et al. claimed through the invention described in patent US9290579B2 a novel biosensing method for creating immobilized protein gradients within three-dimensional (3D) porous scaffolds [45]. One of the notable aspects of this invention is the use of biocompatible materials, including HA hydrogel, in the formation of these scaffolds. The hydrogel’s highly porous structure facilitates the efficient immobilization of proteins, enabling the formation of stable and spatially controlled protein gradients within the scaffold. This controlled gradient is essential for maintaining consistent protein distribution, which improves biosensor stability and allows precise regulation of protein concentrations. Such stability and localization are crucial for applications requiring precise detection and measurement of biological substances, such as medical diagnostics, environmental monitoring, and tissue engineering. By leveraging HA hydrogel, the invention enables enhanced biosensor reliability and efficiency in various detection protocols while also supporting cellular responses or biochemical reactions within the scaffold for broader in vitro and in vivo applications. According to the invention outlined in the claims of the patent, forming biologically active gradients within a 3D scaffold by immobilizing agents is realized through a methodical process of scaffold preparation, agent selection, gradient formation, etc. (Figure 10). By systematically combining these elements (agent type, scaffold preparation, gradient formation, and material choice), the summary of such an invention is as follows:

- Agent selection: The process begins with selecting an appropriate agent, which can include enzymes, cytokines, growth factors, cell binding domains, or cell signaling factors. Each of these agents has distinct functions; for example, growth factors and cytokines can influence cell proliferation and differentiation, while cell-signaling factors are crucial for cell communication. In the context of HA-based hydrogel biosensors, these agents enhance the sensor’s ability to interact with biological targets, providing critical functionality for applications such as cell analysis or biochemical detection;
- Scaffold preparation: The scaffold preparation step involves setting up a 3D porous matrix, which is then contacted with a solution containing the selected agent. A covalent link is formed between the agent and the surface of the scaffold’s pores, creating a stable immobilized gradient. For HA-based hydrogel biosensors, this immobilization strategy allows for a precise spatial arrangement of bioactive molecules, facilitating controlled interaction points within the sensor. This structure is essential for applications that require targeted binding or localized response within the sensor environment;
- Cell gradient formation: After establishing the initial agent gradient, cells can be introduced to create a secondary cell gradient. The cells respond to the immobilized agent (e.g., through chemotaxis or binding interactions), establishing a structured cellular pattern within the scaffold. This functionality is significant in HA-based biosensors, especially for applications in tissue engineering or diagnostics where cellular behavior in response to specific biomolecules needs to be observed or monitored within a hydrogel scaffold;
- Biocompatible material: The scaffold itself can be made from various biocompatible materials, such as silk, collagen, keratin, fibronectin, chitosan, HA, or alginates. HA, specifically, is highly relevant to this invention, as it is widely used for its biocompatibility, bioactivity, and ease of functionalization. HA’s properties enhance the scaffold’s ability to interact with biological entities, making it a valuable medium for creating biosensors with complex and controlled biomolecular gradients.

The method described involves activating the surface of HA hydrogel scaffold to facilitate the binding of agents, such as enzymes. This activation can be achieved through various chemical treatments, which prepare the scaffold for subsequent protein immobilization. Once activated, the scaffold is exposed to a solution containing the desired protein, allowing it to diffuse and create a gradient within the scaffold. This gradient formation is crucial for biosensors as it enables the detection of multiple analytes simultaneously, improving the sensor’s sensitivity and accuracy. Overall, the use of HA hydrogel in this patent demonstrates a significant advancement in the field of biosensors. The material’s biocompatibility, combined with the innovative method of creating immobilized protein gradients, offers a promising approach to enhancing the performance of biosensors. This invention not only contributes to the development of more effective diagnostic tools but also opens up new possibilities for the application of biosensors in various scientific and medical fields [45].

### 4.7. HA Hydrogel-Based Biosensor for Versatile Analyte Detection and Health Monitoring

**Figure 11:**
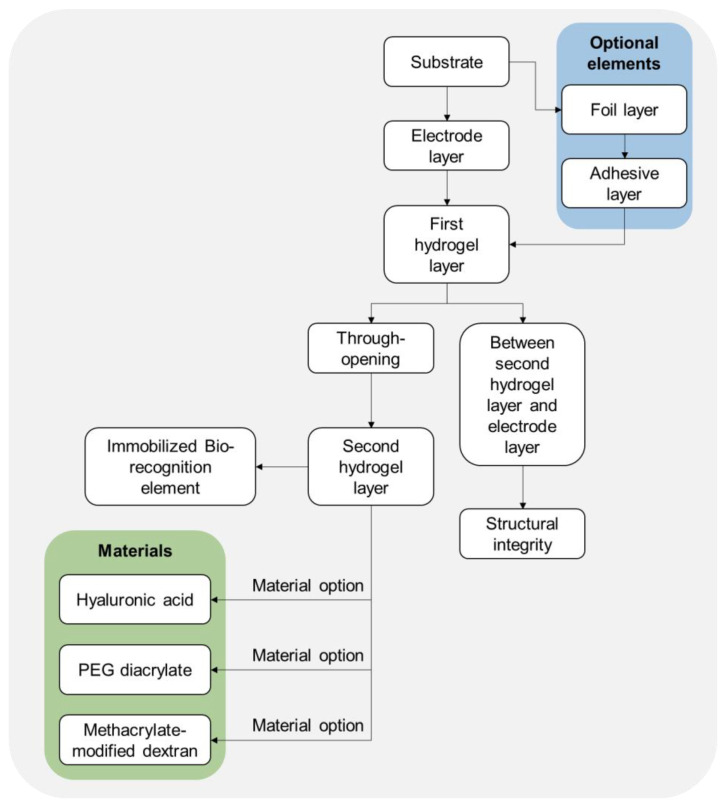
Flowchart representation of the biosensor based on the provided claims through the presented patent. The biosensor features a multi-layered structure where the second hydrogel layer, containing the bio-recognition element, interacts directly with the electrode through an opening in the first hydrogel layer. The first hydrogel layer, potentially made from HA, plays a critical role in maintaining the positioning and functionality of the second hydrogel layer. The flowchart was created and designed from raw data presented in patent US9910005B2 [46].

In 2018, the inventors Di Matteo et al. claimed through the invention the advantages of incorporating hydrogels in biosensing applications [46]. They highlighted the ability of hydrogels to provide near-physiological conditions for proteins, minimize denaturation, and enable the containment of a larger quantity of sensing reagents, ultimately leading to increased sensitivity and signal-to-noise ratio in biosensor technologies. The patent US9910005B2 describes the potential use of HA hydrogel in biosensors as part of the composition of the photo-definable hydrogel membranes. These membranes are utilized for immobilizing bio-recognition elements in biosensors (Figure 11). The HA hydrogel, along with other materials like PEG diacrylate, PEG diamethacrylate, polyproplylene fumerate-co-ethylene glycol, acrylic-modified PVA, methacrylate-modified Dextran, and polyphosphazene, can be used as oligomers and prepolymers in the composition of these membranes. By incorporating HA hydrogel into the composition of the photo-definable hydrogel membranes, the biosensor technology aims to enhance the immobilization of bio-recognition elements on sensor surfaces. This utilization of HA hydrogel, known for its biocompatibility and hydration properties, underscores its potential in providing a suitable matrix for bio-recognition element immobilization in biosensors [46].

### 4.8. Next-Generation HA Hydrogel Biosensor for Sweat Analytes: Comprehensive Design, Manufacturing, and Application Strategies

**Figure 12:**
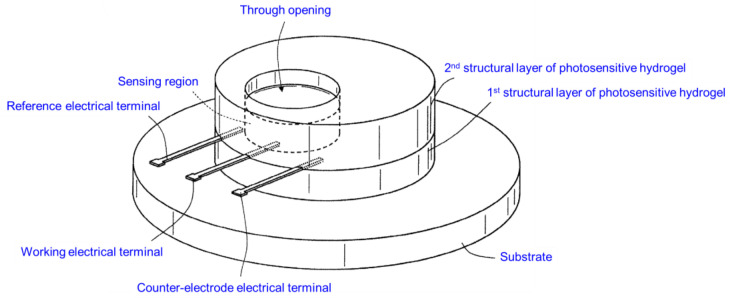
Schematic perspective view of the invented biosensor according to the claimed invention through the presented patents. The scheme was adapted and designed from raw data presented in patents EP3150714B1 and US11116428B2 [47,48].

In 2018, inventors Iuele and Di Palma claimed through European patent EP3150714B1 an innovative biosensor utilizing HA hydrogel for detecting analytes in sweat [47]. This biosensor is designed to provide non-invasive monitoring of glucose and lactate levels, leveraging the unique properties of HA hydrogel to create a sensitive, effective, and user-friendly device for continuous health monitoring (Figure 12). The biosensor comprises multiple structural layers of hydrogel and includes a specialized bioactive region. It features a first structural layer made from a photosensitive hydrogel, a second structural layer also composed of photosensitive hydrogel, and a central bioactive region containing a third type of hydrogel. This bioactive region, which includes embedded enzymes such as glucose oxidase and lactate oxidase, is key for the detection process. The unique configuration ensures that this region is in fluid communication with the external environment, allowing it to receive and analyze sweat fluid effectively. HA hydrogel plays a crucial role in the construction of this biosensor, particularly within the sensing region. The hydrophilic nature of the hydrogel provides an ideal matrix for encapsulating functional enzymes and other biological materials. This environment minimizes the denaturation of these biological elements, thus maintaining their functionality and enhancing the sensitivity and accuracy of the biosensor. The biosensor’s design includes electrodes strategically placed to facilitate accurate readings. A first electrode is coupled to the bioactive region, while a second electrode is spaced apart and positioned on the first structural layer. This arrangement, combined with the hydrogel’s properties, enables the biosensor to efficiently detect and quantify the presence of metabolites like glucose and lactate in sweat. The structured layers of hydrogel ensure that the biosensor is both durable and sensitive, capable of detecting minute changes in analyte concentrations [47].

In 2021, the same inventors further developed their innovation, as described in US patent US11116428B2 [48]. This advanced biosensor utilizes a similar configuration of multiple structural layers of hydrogel and includes a specialized bioactive region. The biosensor is designed to detect glucose and lactate levels, providing a non-invasive method for medical diagnostics. The core of this biosensor features a first structural layer made from a photosensitive hydrogel, a second structural layer also composed of photosensitive hydrogel, and a central bioactive region containing a third type of hydrogel. This bioactive region, which includes embedded enzymes such as glucose oxidase and lactate oxidase, is key for the detection process. The unique configuration ensures that this region is in fluid communication with the external environment, allowing it to receive and analyze sweat fluid effectively. HA hydrogel continues to play a crucial role in the construction of this biosensor, particularly within the sensing region. The hydrophilic nature of the hydrogel provides an ideal matrix for encapsulating functional enzymes and other biological materials. This environment minimizes the denaturation of these biological elements, thus maintaining their functionality and enhancing the sensitivity and accuracy of the biosensor. One of the key advantages of this biosensor is its non-invasiveness. Traditional methods for monitoring glucose and lactate levels typically require blood samples, which can be painful and inconvenient for patients. The use of HA hydrogel in this biosensor allows for the collection and analysis of sweat, eliminating the need for invasive procedures. This makes the device especially suitable for continuous monitoring, providing real-time data on the patient’s metabolic state [48].

Overall, the inventions of these biosensors utilizing HA hydrogel, as detailed in patents EP3150714B1 and US11116428B2, represent a significant advancement in the field of wearable medical diagnostics. They offer a reliable, non-invasive, and user-friendly solution for monitoring important health markers, enhancing the ability to manage conditions like diabetes and ischemia effectively. The innovative approaches outlined in these patents underscore the potential of hydrogel-based technologies in advancing healthcare monitoring systems [47,48].

Finally, it is important to note that both patents EP3150714B1 and US11116428B2 are part of a simple family, indicating their related innovations and that they cover similar inventions in different jurisdictions (Europe and the United States), providing broader protection and reinforcing the inventors’ claims across multiple regions. This also underscores the broad applicability and potential of these technologies in advancing healthcare monitoring systems.

## 5. Challenges and Future Perspectives

Innovative HA-based hydrogel biosensors demonstrate substantial promise for biomedical applications due to HA’s natural biocompatibility and adaptability. However, challenges remain in mechanical stability, electrical conductivity, and sensitivity, which are essential for their effective deployment. Alongside addressing these technical issues, patent analysis offers crucial insights into the evolving landscape, providing direction for innovation and identifying unmet needs in the market.

HA-based hydrogels are inherently soft, making them ideal for biocompatibility but often insufficiently strong for certain applications. To overcome this, reinforcement with materials like reduced graphene oxide and polyaniline has improved compressive strength and elasticity, enhancing wearability for biosensors [14]. Additionally, pseudo-slide-ring hydrogels have shown remarkable stretchability and mechanical toughness, crucial for applications in dynamic, flexible, or wearable biosensors [49]. For HA-based biosensors, effective electrical conductivity is vital for accurate and responsive measurements. Researchers have enhanced HA hydrogels with conductive materials, leading to notable improvements in sensor performance [14]. However, non-implantable biosensors continue to struggle with low sensitivity and slow response times, especially when rapid or high-frequency data collection is required [50]. HA’s natural biocompatibility makes it particularly suitable for in vivo applications, but its interface with biological tissues can introduce high impedance, which affects signal quality [50]. Furthermore, achieving consistent long-term stability is critical for HA-based biosensors designed for extended use. Although some have shown promising stability, maintaining reproducibility remains a key challenge [14].

Patent analysis plays an integral role in identifying trends and innovation gaps in HA-based hydrogel biosensors. By examining patent data, researchers and developers gain insights into dominant research areas, commonly addressed challenges, and emerging applications. For instance, patent filings reveal a focus on glucose monitoring and sweat analyte detection, highlighting the demand for non-invasive and wearable biosensing solutions. Through patent analysis, the progression of HA-based hydrogels can be mapped to see where innovation in mechanical reinforcement, biocompatibility, and electrical performance is advancing or lagging, thereby informing targeted research and development efforts. Furthermore, it identifies regional strengths, as seen in the high concentration of United States patent filings, and helps guide international collaboration between academia and industry to accelerate biosensor advancements.

While HA-based biosensors are advancing through both technical research and intellectual property developments, further work is needed to refine the balance between mechanical durability, electrical performance, and biocompatibility. Future research should explore advanced crosslinking methods, innovative reinforcement materials, and expanded patent landscapes to ensure regulatory alignment and market readiness. Addressing these challenges will be critical for the successful deployment of HA-based biosensors in clinical and wearable applications, positioning them as pivotal tools in the future of personalized health monitoring.

## 6. Conclusions and Outlook

In summary, this patent overview demonstrates how HA-based hydrogels are driving innovations across the medical device and diagnostic sectors, underscoring their critical role in advancing healthcare through novel biosensor technologies. HA-based hydrogels, with their inherent biocompatibility and tunable properties, have shown significant promise, particularly in glucose monitoring and other areas of health diagnostics, where precision and real-time monitoring are essential. Their improved mechanical properties and modifiable electrical conductivity enhance their applicability in biosensors, opening up opportunities for developing sophisticated, efficient biosensing systems with potential use in a variety of clinical and wearable settings.

Regulatory and commercialization challenges remain substantial, as ensuring safety and efficacy in long-term clinical use is paramount. Future research should prioritize collaborations with regulatory bodies to develop standards for HA-based biosensors, ensuring these devices meet stringent healthcare requirements. Likewise, partnerships with industry stakeholders will be critical to streamline commercialization processes, from scaling production to developing cost-effective manufacturing techniques, thereby accelerating market entry.

The future scope of HA-based hydrogel biosensors extends beyond conventional health monitoring. Emerging applications may include personalized health diagnostics, real-time sweat analysis for athletic performance, and in situ monitoring of wound healing, capitalizing on HA’s natural compatibility with biological tissues. Furthermore, HA-based hydrogels have potential as implantable biosensors for continuous, minimally invasive monitoring in various therapeutic areas, such as cardiovascular health and metabolic disorders. Continued interdisciplinary research in biomaterials science, biomedical engineering, and nanotechnology will be crucial in realizing these applications, transforming HA-based hydrogel biosensors into versatile tools that could reshape personalized healthcare and preventative medicine.

## Figures and Tables

**Table 2:** Top 10 CPC codes as a function of patent count and HA-based hydrogel applications/formulations in the biosensor area.

Patent	Publication	Title	Ref.
US7432069B2	2008	Biocompatible chemically crosslinked hydrogels for glucose sensing.	[40]
US8367109B2	2013	Microbes encapsulated within crosslinkable polymers.	[41]
US8608922B2	2013	Biosensor for continuous monitoring of metabolites and proteins and methods of manufacture thereof.	[42]
US9011330B2	2015	Implantable vascular system biosensor with grown capillary beds and uses thereof.	[43]
US9244064B2	2016	Use of hydrogels for biosensors having elevated sensitivity	[44]
US9290579B2	2016	Covalently immobilized protein gradients in three-dimensional porous scaffolds.	[45]
US9910005B2	2018	Biosensor	[46]
EP3150714B1 *	2018	Biosensor for sensing analytes in the sweat and manufacturing method thereof.	[47]
US11116428B2 *	2021	Biosensor for sensing analytes in the sweat and manufacturing method thereof.	[48]

* Both patents, EP3150714B1 and US11116428B2, are part of a simple family of patents, indicating that they cover similar inventions in different jurisdictions (Europe and the United States), providing broader protection and reinforcing the inventors’ claims across multiple regions.

## Data Availability

The data presented in this study are available within the content of this article.
